# Supplementary material for: Are carbon emissions trading and green financial instruments synergistic? -Comprehensive quantitative research based on content analysis
Source: PLoS One. 2024 Mar 7;19(3):e0298601. doi: 10.1371/journal.pone.0298601 (PMC10919601; doi:10.1371/journal.pone.0298601)
Supplement: S1 Data — (ZIP) [file pone.0298601.s001.zip › Data/地级市Z_F工作报告.zip!/║┌┴·╜¡/║┌║╙╩╨/└┤╫╘╣1⁄2╓┌║┼í╛┬φ┐╦╩2╛▌═°í┐_2015.docx]

各位代表：

现在，我代表市人民政府，向大会作工作报告，请予审议，并请列席会议的同志提出宝贵意见。

一、2014年工作回顾

2014年，我们在省委、省政府和市委正确领导下，紧紧围绕稳增长、促改革、调结构、惠民生，积极应对经济下行压力，努力化解不利因素影响，主动对接“五大规划”，继续打好“三大攻坚战”，实施“五大工程”，经济社会实现平稳健康发展。预计全市生产总值424亿元，增长9%。全社会固定资产投资245亿元，增长11.7%。社会消费品零售总额95亿元，增长13%。公共财政收入26.4亿元，增长14.2%，其中，市直8.2亿元，增长18.3%。城镇居民人均可支配收入、农村居民人均纯收入均增长10%。万元地区生产总值综合能耗下降3.4%。

——产业项目势头强劲。全力推前期、促开工、抢进度，项目和园区建设全面提速。80项市级重点产业项目开复工77项，完成投资112亿元，增长37%，其中17项省重点推进产业项目开复工16项，完成投资37亿元。库南铅锌矿、盛辉林板一期、中俄黄金子仓储物流等30个项目建成投产，中铝力拓整装勘查、翠宏山铁多金属矿、争光岩金矿、谷神大豆、恒大粮油、弘昊物流等项目进展顺利。狠抓项目谋划和储备，提高成熟度和对接度，新谋划的350项产业项目一半以上达到建议书或可研程度。园区载体功能进一步提升，全市各园区基础设施投资4.5亿元，新开发面积5.7平方公里，开复工项目74项，完成投资47.3亿元，黑河(北安、孙吴)健康产业园特色更加凸显，吸引了科迪食品、修正药业、佐源集团等一批投资亿元以上大项目入驻，合作区中小企业创业中心成为科技型、创新型企业发育平台。加大招商引资力度，与阿州首次共同举办中俄双子城展览会，组织参加了首届中俄博览会、民企龙江行等重大招商活动，引起国内外客商广泛关注，落实项目291项，到位资金246.3亿元，增长33.1%。强化要素保障，金融机构投放企业贷款增长21.8%，新增规模以上工业企业7户，工业实力不断增强。非公经济税金25.4亿元，占全市税收的79.6%。加快科技成果转化，新增国家级高新技术企业2户，省级科技型中小企业20户，高新技术产业产值增长30%。

——现代农业加快发展。坚持“四化引领、四点支撑、四措并举”，推进现代农业综合配套改革，增强农业发展活力。加快土地流转，适度规模经营面积达到1200万亩，适宜耕地全部实现规模经营。以农机化提升农业现代化，田间综合机械化程度达到98%。粮食生产获特大丰收，再创历史新高。调整优化种植结构，主栽作物品种实现科学轮作，大力发展玉米、薯类等优质高效作物，扩大蔬菜、杂豆等经济作物规模，农业综合效益进一步提升。加快推进水稻北扩，面积达到40万亩，举办了中国黑河高纬寒地水稻发展论坛。发挥寒地资源和生态优势，主打绿色食品品牌，国家级绿色食品标准化基地达到280万亩，绿色(有机)、无公害农产品种植面积850万亩，认证产品71个。强化龙头企业牵动，规模以上加工企业达到70户，农产品加工转化能力进一步提高。大力发展畜牧业，标准化规模养殖小区达到116个，中兴牧业罕达汽、西峰山牧场通过省级现代示范养殖场验收，新建霍龙门牧场完工，实现了奶牛散养向集中规模养殖转型。打造黑龙江网箱养鱼、五大连池矿泉冷水鱼养殖示范区，省部级健康养殖示范场达到30个。加快发展林业特色产业，新增蓝莓、忍冬等生态经济林1万亩，北药种植面积达到58万亩，嫩江中药饮片项目二期投产。新型经营主体进一步壮大，各类农民专业合作社达到1249个，21个合作社获国家和省级示范社、百强社。现代农机专业合作社达到133个，5个合作社被认定为省级规范社。农村土地承包经营权确权登记试点稳步实施，北安石泉镇国家级、嫩江科洛镇省级以及市级试点全面展开。

——对俄合作深度拓展。积极应对俄经济形势变化，引导企业调整结构，对外贸易运行质量不断提高，对俄经贸合作态势良好，多层次、宽领域、全方位的对俄交流格局基本形成。对俄通道建设得到国家、省高度关注和大力支持，纳入“中蒙俄经济走廊”龙江陆海丝绸之路经济带规划，黑河在沿边开放中的战略地位更加巩固。跨境产业合作层次不断提升，阿穆尔—黑河边境油品储运与炼化综合体项目通过国家发改委核准，中俄东线天然气管道境外工程开工，年产60万吨水泥粉磨站当年开工、当年建成，境外土地开发105万亩，回运大豆2.1万吨。举办了第七届中俄林业生态建设学术论坛，与阿州就共同开展野生动物资源及栖息地保护达成共识。和兴物流加工产业园、北丰农业产业园等境外园区建设加快，已成为国内企业进入俄市场的有效载体。对俄人文交流不断深化，“两国一城、共同繁荣”理念深入人心。对俄交往领域进一步拓展，与萨哈(雅库特)共和国涅留恩格里区正式缔结友好城市。举办中俄教育交流活动40余次，参与师生2.4万人，中俄青少年文艺演出和国际夏令营活动得到国家教育部关注。举办了中俄篮球、乒乓球友谊赛，互派团组参加对方举办的运动会。“三八”妇女节、“六一”儿童节互访和横渡黑龙江三项活动影响力不断提升，两地间人文交流更加紧密，经贸往来更加活跃，民众关系更加融洽。服务环境不断改善，取消减免11项口岸收费，年降费5000万元，对口岸和铁路大宗货物运输实行优惠价格。开展重点行业专项治理，互贸区消费环境进一步优化。互市贸易监管点通过验收，交易点试运营，市民享受到政策红利，促进了互贸区繁荣发展。

——新兴业态持续升温。挖掘地域特色资源，培育新经济增长点，提升现代服务业水平。文化产业成为新亮点，成功举办第五届中俄文化大集，辐射中国内地和俄远东地区，成为中俄边境地区文化交流典范。嫩江墨尔根古道驿站博物馆开馆，北安新文化广场主体完工，北安获全国文化先进县。旅游产业健康发展，边境游企稳回升，出境游强劲增长，全市接待旅游者、旅游收入分别增长12.8%和12%。五大连池风景区通过世界生物圈保护区、世界地质公园评估，入选全球首批世界自然保护联盟绿色名录。五大连池山口湖风景区、北安庆华军工遗址博物馆分别晋升国家4A级、3A级景区。乡村游展现特色魅力，爱辉区新生乡入选“全国100个值得记忆乡愁的地方”，新生村入选首批“中国少数民族特色村寨”。与俄方共同开发黑河—布市乡村之旅，与哈尔滨、伊春和阿州成立了“3+1”跨境区域旅游联合体。成功举办“飞越冰河、超越梦想”汽车飞越黑龙江活动。培育“新四型经济”，养老产业步伐加快，市城乡老年活动中心正式启用。珠宝玉石产业影响力逐步扩大，举办了观赏石展，10余省观赏石协会的200余位客商参展。试车经济蓬勃发展，来黑河试车的生产研发企业达到75户。碳汇经济稳步推进，完成碳汇造林2500亩，募集碳汇资金257万元。

——基础设施建设取得突破。致力于打基础、利长远，加大交通、水利等重大设施建设力度，为长远发展奠定了坚实基础。推进多年的跨境交通项目前期取得重大突破，黑龙江大桥上升到中俄国家层面推进，举行了13次部委、省州、市州会谈，正在筹建中俄联合公司。跨江空中索道取得实质进展，前期工作加快推进。五大连池民用机场正在进行可研审批，建成后将形成“一市两场”航空网络，立体化大交通格局将进一步完善。嫩(泰)高速公路嫩江互通项目开工，沾五公路升级改造竣工通车，北安铁路跨线桥100天建成。水利投资规模再上新台阶，全市完成投资7.9亿元，是上年的2.5倍，全市单体投资最大的黑河水利防护工程开工建设，国家战略性水利工程三江治理全面启动，我市212.4公里堤防纳入，总投资26.2亿元，黑河城区段是三江治理工程中唯一按“百年一遇”标准批复建设的堤防，体现了国家对黑河的重视和关怀。全面开展灾后水毁工程修复，完成投资2.8亿元，开工项目超百项，修复国省干线公路33公里、农村公路160公里、桥梁17座，黑干、嫩干和中小河流堤防修复工程开工，高质量完成灌涝区修复，为灾后夺取粮食丰收提供了重要保障。

——城市建设提档升级。把“三城同创”作为提升城市品位、改善人居环境的重要载体，市民幸福感和城市美誉度不断提升。国家卫生城创建取得突破，全民动员，合力攻坚，顺利通过国家评估组验收，近期有望获批。园林城通过省级初审，环保模范城加快推进。完善城乡规划编制体系，城市总体规划通过省审查。加大城市基础设施投入，货运浮箱固冰通道连接路启动建设，市区西部3条道路开工，四中北侧道路东段完工。完成市区323处架空管线落地改造。新建绿荫停车场5处，新增便民自行车停放架60处，改造小区进出道口90处。污泥处理厂主体工程、生活垃圾处理厂渗滤液无害化处理工程完工。改造供热管网26公里、供水管网4.9公里，撤并小锅炉房15个。完成130栋楼体亮化设施大修。开展绿化大会战，完成市区31条街路、11处节点栽植补种，新增绿地面积8.9公顷，绿化率达到35.4%。加大环境整治力度，拆除违建1.5万平方米。改善居住条件，全市开工建设保障性安居工程7585套，改造农村泥草(危)房7034户。市区南部棚户区动迁完成98%。廉租住房实物配租260户，发放租赁补贴5086户，维修改造老旧住宅楼房112栋，改造提升住宅小区30个。开展“精细化管理提升年”活动，全面铺开限时保洁制，城市管理长效机制进一步完善。大力保护生态环境，全市完成造林绿化37.3万亩，森林抚育43.7万亩，退耕还林还草还湿10万亩。天然林保护政策争取工作得到国家相关部门支持，森林生态系统定位观测研究站通过国家审批。

——民生保障更加有力。恪守为民之责，倾力解决民生难题，“十六件民生实事”全部完成，荣获“全国首批民生改善十佳典范城市”。城镇新增就业2.1万人，安置高校毕业生1680人，应届毕业生初次就业率82%，城镇登记失业率3.72%，低于省定目标0.88个百分点。加大教育投入，建成教育管理公共服务平台和教育资源公共服务平台，新建、改扩建公办幼儿园9所，义务教育达标学校达到85所，黑河技师学院获省政府批准，五大连池省部共建国家现代农村职业教育改革试验区试点县通过中期评估。高考实现“三连增”，全市文理科一本、二本上线率分别提高4.7和14个百分点。构建“安全和谐校园”，市区学校视频监控全部纳入公安机关监控系统。大力发展医疗卫生事业，为市直三家医院购置了先进医疗设备，建设了远程会诊及远程医疗教育系统。全市公办基层医疗卫生机构基本药物全部实行网上集中采购、零差率销售，基本药物制度覆盖率100%。提前一年完成独生子女父母奖励费清欠任务。妇女儿童权益保护工作进一步加强。打造“动感城市”，承办冰雪汽车挑战赛、首届大众健身操争霸赛等体育赛事80余项。第五届运动会成功举办，是历届规模最大、人员最多的一次体育盛会，展示了全市人民顽强拼搏、勇于争先的精神风貌。档案、地方志等工作也取得新成绩。社保水平不断提升，城市低保标准每人每月提高到440元，农村低保标准每人每年提高到2700元。市直城镇职工基本医疗保险住院医疗费最高支付限额由8万元提高到10万元，大病医疗补助由10万元提高到15万元，大额医疗保险报销比例由75%提高到80%。市直在全省率先启动三亚、哈尔滨异地就医即时结算试点。新农合参合率达到99.97%。企业退休人员基本养老金每月增加222元，工伤人员伤残津贴、遗属生活费和护理费标准每月提高214元，市区环卫保洁人员工资每月增加200元。社区建设走在全省前列，城市达标社区达到56个，农村社区启动建设462个，爱辉区12个城市社区全部达到精品以上标准，爱辉区、五大连池青山街道办事处、北安百盛社区成为全国第二批和谐社区建设示范单位。爱辉区荣获全国民族团结进步模范集体。安全生产形势保持稳定，连续17个防期未发生重特大森林火灾。深入开展“红盾护农”、“春雷行动”。食品药品安全监管进一步加强。强化人防执法监督，全民防震减灾意识明显增强。信访形势总体平稳。军政军民融合发展，边境秩序保持稳定。

——政府效能全面提升。开展党的群众路线教育实践活动，坚持问题导向，实施11项重点专项治理，深入整治“四风”问题。建立健全督办落实、调查研究等21项制度，推进作风建设机制化。对市直机关事业单位办公用房进行清理，清退面积8773平方米。严格控制支出，市直“三公”经费下降50%以上。坚持依法行政，主动接受人大依法监督和政协民主监督，177件建议和提案全部办结。修订完善了重大决策社会稳定风险评估、听证和专家咨询论证等制度，建立健全决策、执行和监督机制，增强了政府执行力和公信力。深入推进行政审批制度改革，取消下放135项行政审批事项，启动工商登记制度改革。严格落实党风廉政建设“一岗双责”，强化专项治理、跟踪审计，政府建设进一步加强。

各位代表，过去一年，我们在科学发展的道路上迈出了坚实步伐，成绩来之不易，是省委、省政府和市委正确领导的结果，是全市上下齐心协力、埋头苦干的结果。在此，我代表市人民政府，向全市各级人大代表、政协委员和各民主党派、工商联、无党派人士、人民团体，向中省直各单位和人民解放军、武警官兵，向全市人民，表示衷心感谢，并致以崇高敬意!

我们在看到成绩的同时，也要清醒看到工作中存在的问题。受国内市场影响，煤炭产量和价格双下降，冶金行业富矿减少，新建项目支撑作用还没有充分显现，我市煤炭、冶金行业增加值占规模以上工业的47%，直接影响了工业增速。俄罗斯经济形势变化，一般贸易出口受到严重冲击，影响了边境旅游和消费。财政减收增支因素增多，保障改善民生的压力加大。政府行政效能、依法行政水平还有很大差距。对这些问题，我们将在今后工作中，下决心、下大力气加以解决。

二、2015年目标任务

正确认识形势、研判走势是做好各项工作的基础和前提。中央经济工作会议从经济增速、发展方式、经济结构、发展动力等方面，揭示了新常态的基本特征，是今后一个时期科学发展的基本遵循。我们一定要深刻审视市情，牢牢把握发展的阶段性特征，在新方略中抢先机，在新常态下有新作为，加快建设“一都三地、四位一体”魅力黑河。

——认识新常态，保持工作定力。辩证分析新常态对黑河的影响，既找准优势、深挖潜力，又扬长避短、有所作为。应该看到，矿产资源开发项目审批时间长、见效慢制约矿业富市进程，煤炭行业限产和价格低迷的影响还在持续，农业生产成本高、比较效益低制约农业产业化发展，对俄贸易和投资还有一些不确定因素。但更要看到，我市在对俄合作、生态建设、城乡发展、资源深加工等方面的巨大潜力，特别是国家沿边开放、俄罗斯远东开发，给黑河发展带来的重大政策红利。我们一定要抢抓机遇、保持定力，以高于全省的速度加快发展，实现跨越赶超。

——适应新常态，增添发展动力。在新常态下实现新发展，结构优化、动力转换的要求尤为迫切。我市农业种强销弱，特色不突出；工业总量小，内部结构不合理；消费受人口规模制约，第三产业发展氛围不够。我们要做大存量，延伸产业链，提高产业层次，加强与大企业战略合作，促进科研成果引进和优化升级，推进现有企业发展壮大。同时，要形成新增量，打造新的经济增长点，大力发展新产品、新业态、新商业模式，激活未来竞争的新能量，为经济增长注入源源不断的新动力，实现有质量、有效益、可持续的发展。

——引领新常态，坚持正确方向。遵循经济社会发展客观规律，立足黑河实际，牢牢把握未来发展方向。我们要始终秉承市五届人大一次会议确定的“一个发展理念”，坚持生态发展、实现绿色崛起不动摇，发挥生态这个最宝贵资源和最大发展优势，全力守住非转基因这片净土，使生态优势助推经济发展，绿色崛起成果惠及广大群众。要始终把握市五届人大二次会议确定的“一个工作方针”，坚持“六个工作原则”，强基础、利长远、求创新、促发展，把心思和精力用在干事创业上，不脱离实际，不好高骛远，一张蓝图绘到底，一以贯之抓落实。要始终追求市五届人大三次会议确定的“一个根本目标”，推动“四个发展”，惠民生、增福祉，越是经济下行压力大的时候，越要把民生工作放在突出位置，既多做雪中送炭的“暖心事”，又多做提高生活品质的“舒心事”，让全市人民吃得放心、住得舒适、行得安全，过上更加幸福美好的生活。

今年是全面深化改革的关键之年，也是全面完成“十二五”规划的收官之年。我们要全面贯彻党的十八大、十八届三中、四中全会精神，落实中央、省委工作部署，按照市委五届八次全会要求，持之以恒打好“三大攻坚战”，实施“六大提升工程”，稳中求进、进中求好、好中求快，促进经济持续健康发展，社会和谐稳定。

主要预期目标是：全市生产总值增长8%。全社会固定资产投资增长12%。社会消费品零售总额增长12%。公共财政收入增长10%。规模以上工业增加值增长7%。城镇居民人均可支配收入、农村居民人均纯收入均增长10%。万元地区生产总值综合能耗下降2%。

(一)打好产业项目攻坚战。坚定不移育产业、上项目、扩投资，全力以赴抓谋划、推进度、落责任，全市安排重点产业项目89项，年度投资112亿元。提高项目谋划专业水准。高质量编制“十三五”规划，谋划一批打基础、利长远的重大项目。根据我市资源禀赋、区位条件、产业配套等特点，邀请国内外大专院校、科研院所帮助谋划项目，提高项目谋划专业化程度。做深项目前期工作，重点项目均达到建议书或可研程度。广泛寻求战略合作。新常态下引资引智竞争将更加激烈，要继续坚持“九个更加注重”，紧盯招商线索不放，积极寻求战略合作，以资源换产业，整体开发矿产和旅游资源，全力推进“五大产业”壮大和“新四型经济”发展。加强对俄科技合作，依托中俄国际科技产业城、中俄林业科技合作园区、境外产业园区等平台，促进科技合作成果转化。注重与资本市场衔接，扶持具备条件的企业在“新三板”挂牌，设立政府产业基金，提高政府性担保机构融资能力，扶持现有企业做大做强。在引进资金、项目的同时，注重引进人才、技术和品牌，做到引资引智并举。招商引资到位资金增长20%。狠抓项目开工和投产达效。严格落实包保责任，新项目快开工快建设，建成项目快投产，投产项目快达效。重点产业项目开复工率和投资完成率均达到90%以上。金融机构为企业投放贷款增长10%以上，向上争取到位资金增长10%以上。高标准完善园区功能，做好“筑巢引凤”文章，抓好黑河进出口加工产业园、黑河(北安、孙吴)健康产业园等特色园区建设。

(二)打好重大基础设施建设攻坚战。抓住国家和省政策机遇，加快关系全局、影响深远的重大项目建设，为经济社会发展提供保障。推进交通设施建设。全力配合省交通厅，积极协调俄方，做好黑龙江大桥、跨江空中索道项目前期各项准备，力争年内尽快开工。全力推进五大连池民用机场前期，上半年机场及附属工程开工。加强与央企、民企合作，推进北五铁路建设和北黑铁路改造升级。实施水毁公路修复，开工市管6条国省道重建项目。强化水利工程建设。加快建设黑河水利防护工程，实施好三江治理黑河城区段堤防达标工程。推进嫩干水毁修复和逊克、孙吴、爱辉区黑干堤防应急达标工程建设。实施黑河城区、逊克城区、爱辉区护岸工程。开工7座水库除险加固工程，实施7条中小河流治理。建设旱田节水灌溉和10个灌区工程，新增旱田节水灌溉面积10万亩。加快粮食仓储设施建设。坚持合理布局、统筹安排，新增仓容15万吨，新建烘干塔8座。鼓励社会多元主体建设仓容和烘干设施，引导组建粮食仓储烘干服务合作社，提高粮食仓储烘干能力，确保粮食安全。

(三)打好城市建设攻坚战。完善黑河城市功能，打造城市特色，巩固国家卫生城创建成果，全力争创国家园林城和环保模范城。强化规划引导。以新一轮城市总体规划为依据，规划建设“一带三区十节点”沿江生态景观，打造集生态、文化、亲水、休憩为一体的城市体验新空间。“一带”为百里界江、山水环绕的城市沿江风光带，“三区”为以群山密林为基底、保护开发为特征的西部山林区，以黑龙江为基底、升级改造为特征的北部滨水区，以湿地为基底、生态开发为特征的东部湿地区，打造卧牛湖、中俄民族风情园、振边酒厂等十个景观节点。结合黑河水利防护工程建设，高水平实施沿江公园改造，突出大黑河岛观光娱乐、运动体验、休闲健身功能，打造国际商贸文化旅游岛。完善市政设施。推进货运浮箱固冰通道连接路、四中北侧道路西段、老通江路3条道路建设，实施楼体通信管线归集整理、路灯安全加固工程。改造火车站站前广场。开工建设南部棚户区一期工程，两年内完成1074户回迁居民安置。加快“三供三治”项目建设，实施小区供水管网改造，开展地下管网现状普查，制定整体改造方案，力争用5～10年时间完成改造任务。实施生活垃圾处理厂填埋区膜覆盖工程，新建移动式生活垃圾压缩站6座。探索公共资源配置市场化，利用PPP和特许经营等路径，加快建设供热、供气、供水、城市交通等公共资源项目。推进园林绿化。优化绿化格局，对22条街路进行绿化，断点街路和部分小区进行补植，10个单位庭院进行绿化改造，将208个小区纳入绿化管护体系。提升绿化品位，新增五色草和立体绿化景观，建设白桦林公园，举办第二届花卉节。市区新增绿地面积33.4公顷。力争国家园林城通过验收。加强城市管理。推动智慧城市建设，提高城市信息化水平。加强行政执法与公安部门联动，完善联合执法机制，提高应对突发事件处置能力。实施道路保洁分类管理，提高机械作业水平。加强物业管理，提升服务质量，扩大物业管理覆盖面。对机场路全线、202国道连接线、黑爱公路、黑呼公路5公里以内的违章建筑和户外广告，进行清理整顿，实施规范管理。同时，以黑河城区建设管理水平的提档升级，以市带县、以城带乡，实现城乡互动发展，精心打造富有时代气息的现代宜居城市，充满田园风情的美丽乡村，让人与山相依、与水为伴，享受惬意生活。

(四)实施高纬寒地特色农业提升工程。突出“两大平原”现代农业综合配套改革，用现代发展理念引领农业，实现“四个转变”，促进农业增效、农民增收。建基地调结构。突出寒地黑土优势，建设非转基因大豆、强筋小麦、优质玉米、功能水稻和林果、北药生产基地，发展绿色(有机)、无公害农产品，开发功能性稻米、健康食品。绿色(有机)食品认证面积达到300万亩。蔬菜种植面积达到12万亩，农产品出口基地面积达到15万亩。主动对接省亿亩生态高标准农田建设项目，搞好规划，争取资金支持。实施畜牧业发展三年规划，推动畜牧养殖大发展，高品质规模化奶牛场达到60个。做大中兴牧业，奶牛存栏达到2.5万头，推进嫩江奶牛规模养殖场二期，加快爱辉区和孙吴乳制品深加工等项目建设。切实做好动物重大疫病防控工作。扩大名优新品种渔业养殖规模，水产品产量增长11%。壮龙头育产业。坚持走产业化道路，支持谷神大豆、恒大粮油等企业发展，扶持本土企业做大做强，力争新增年产值千万元以上企业5家。完善利益联结机制，促进龙头企业与农民专业合作社融合，共同发展壮大。延长产业链条，开发市场前景好的新产品，提高农产品转化率和增值率。打品牌拓渠道。坚持引进知名品牌与突出本地特色结合，做大做强爱辉区“瑷珲”山珍、嫩江“食佳”面粉、北安“宜品”乳业、孙吴“长乐山”饮品等品牌，培育扶持黑河“亚欧双子城”大豆、北安“龙达”种业等品牌。发挥各级供销系统主渠道作用，建立完善连锁经销网络体系。加快电子商务平台建设，在北京、上海等大中城市新建直销实体店。加强农产品质量监控体系建设，建立生产记录、产地证明和产品准出追溯制度。强主体促改革。培育壮大新型经营主体，扶持家庭农场和种粮大户，新型经营主体增长10%。推进农机合作社规范化建设，新增省级规范社10个、市级示范社30个。总结试点经验，稳步推进农村土地承包经营权确权登记。强化县级农业技术推广机构和乡镇农业综合服务站建设，完善农业信息网和信息服务平台。

(五)实施对俄合作转型提升工程。巩固对俄合作地位，建设沿边开发开放区域中心城市；在“两国一城、共同繁荣”理念下，把“中俄双子城”打造成“中蒙俄经济走廊”龙江陆海丝绸之路经济带上的中俄友好交流典范；黑龙江省对俄自由贸易区的重要节点。深化人文交流。以共同举办反法西斯胜利70周年纪念活动为契机，提升第六届中俄文化大集层次，扩大横渡黑龙江活动影响力，推动“三八”妇女节、“六一”儿童节和民间互访活动，继续深化与阿州及布市教育、医疗、体育等领域交流。同时，扩大与萨哈(雅库特)共和国和克拉斯诺亚尔斯克、伊尔库茨克等俄远东城市友好交往，用人文交流引领经贸合作。扩大贸易规模。加快发展国际物流产业，打造中俄能源资源合作区域物流集散中心。坚持“引进来”与“走出去”并重，联手大连、东莞等贸促会，吸引更多企业入驻黑河，经黑河口岸过货。继续与阿州共同举办中俄双子城展览会。发展边境小额贸易，扩大本地产品出口份额和境外农产品回运规模。积极发展电子商务，引进国内大企业和电商企业开展对俄跨境物流业务，扶持本地企业创建跨境平台。深化投资合作。主动对接“中蒙俄经济走廊”龙江陆海丝绸之路经济带规划，力争重大建设项目在黑河布点。加快合作项目建设，阿穆尔-黑河边境油品储运与炼化综合体项目开工，丰泰公司境外汽车展销及售后服务中心项目投入使用，推进利源达集团境外机电营销网络项目，扩大在俄市场份额。加快在俄铁矿、煤矿开发，力争取得实质进展。加快园区发展。积极谋划申报国家级跨境经济合作区，打造跨境贸易和产业合作新载体，促进跨境产业融合发展。继续推进和兴物流加工产业园、北丰农业产业园建设，加快阿州境外工业园区前期。新建农产品仓储批发销售区，争取年内投入使用。优化服务环境。开展“口岸服务年”活动，继续整顿口岸通关环境，提高工作效率和服务水平，加强对俄沟通协调，保证客货畅通。继续开展创建优良消费环境活动。完善口岸基础设施，旅检民贸大厅交付使用，实现口岸旅贸分检。

(六)实施旅游形象提升工程。突出高端定位，提升旅游竞争力，打造中俄跨境旅游合作区。旅游收入增长11%。开拓客源市场。加强与全联旅游商会合作，完善智慧旅游公共服务平台，强化宣传、推广、交易功能，打造覆盖全国的旅游智慧交易推广网络，完成百万游客交易量。加强与国家和省级媒体合作，通过央视《走遍中国》栏目、北上广等28个一类城市机场电视、全国高铁2670组列车视频、黑龙江龙广之声，全面展示黑河旅游形象，提高知名度和影响力。巩固东北客源地，强化省内、周边城市及市域内旅游合作，拓展国外及环渤海、长三角、珠三角等高端旅游市场。打造精品景区。建设五大连池风景区国际中医矿泉健康园区，开发卧虎山等新景点，开工SPA城、焦得布森林公园等项目，创建国家生态旅游示范区。完善瑷珲古城旅游区、红河谷国际滑雪场、孙吴胜山要塞等重点景区功能，推进五道豁洛岛开发和五大连池、卧牛湖自驾车营地建设。开发精品线路。整合优势资源，合理设计旅游线路和产品组合。立足市区、五大连池景区“两大核心区”，培育五大连池火山矿泉-黑河民俗风情-俄布市异国风光“黄金旅游带”，带动市域内周边游。拓展边境旅游新线路，完善黑河-贝加尔湖生态之旅，打造跨境自驾游旅游产品。开通黑河至韩国旅游线路。加强与省外旅游企业合作，提高边境风情游、康疗养生游、跨境自驾游、森林生态游影响力。优化旅游环境。开展“旅游服务质量提升年”活动，强化旅游监管。完善旅游服务平台，建设黑河游客中心，成立旅游信息中心。引入星级饭店退出机制，维护消费者权益。

(七)实施文化产业提升工程。突出欧陆、民俗、知青等个性化文化元素，运作城市文化资本，提高文化软实力，打造中俄文化艺术交流示范区。搭建对俄文化产业平台。规划建设中俄文化创意产业园，筹建中俄文化博览交易中心，建设中国书画艺术院黑河分院，推进黑河学院与俄苏里科夫美术学院合作，扩大俄艺术品展销规模，促进对俄文化交流向文化产业合作拓展。与阿州共同打造“十里龙江文化风情游”，开发黑龙江沿线岛屿，突出中俄风情演艺、中西餐饮、影视娱乐等文化元素，游界江、登两岸、赏四岛风光，建设“一江两岸四岛文化旅游圈”。开发文化产业项目。谋划文化产业发展思路，制定文化产业发展总体规划。加快中俄民族风情园建设，推进中俄画家村、北方影视城项目。积极推进瑷珲古城整体开发，加快建设瑷珲国家森林公园、知青馆接待中心，完成瑷珲海关陈列馆布展。力争莫日根国际狩猎场投入使用，完善刺尔滨鄂伦春族原始部落体验区功能。开工森林湿地馆。筹建红玛瑙文化产业园。突出冰雪资源特色，开发冰雪文化项目。整合地域文化资源。构筑文化符号，完善博物馆群，传承文化记忆。延续历史文脉，建设爱辉区少数民族文化走廊、知青村和爱辉区、逊克俄罗斯族特色村寨。提高文化遗产保护意识，加强摩苏昆等非物质文化遗产保护传承。鼓励创作优秀文化作品，支持民间工艺品发展。培养本土文化人才，引进经营管理人才，促进文化产业繁荣。

(八)实施生态建设提升工程。珍惜良好生态，保护美好家园，让蓝天白云、碧水青山成为黑河的靓丽名片。强化森林资源保护。力争天然林保护纳入国家政策支持。加大造林绿化力度，完成造林绿化18.2万亩，森林抚育42.4万亩，冠下补植3万亩。继续开展“一退三还”行动，完成退耕还林还草还湿11万亩。严厉打击侵占林地、毁林毁草毁湿和破坏野生动物资源行为。巩固碳汇造林成果，提高社会认知度，推进碳汇林业发展。完善森防网格化指挥体系，严格野外火源管理，确保不发生重特大森林火灾。开工森林生态站、北部重点林区森林扑火能力建设工程，林业信息中心一期投入运行。加强与阿州合作，推进野生动物跨境自然保护区前期。提高环境质量。加强空气质量监测，加快淘汰黄标车，抓好秸秆综合利用，减少大气污染。强化噪声监管，解决交通、施工和生活噪声扰民问题。加大水土流失治理力度，继续开展河道采砂专项治理。落实取水许可和水资源有偿使用制度，完善水资源管理信息系统。加大农业面源污染防治力度，实施“黑土保护”和“沃土工程”，推广测土配方施肥、秸秆还田、增施农家肥等措施，加快中低产田改造，加大深松整地力度，规范农业投入品使用管理，降低耕地化肥、农药施用强度，做好农药包装废弃物回收工作。抓好节能减排工作。实施绿色矿山试点项目，创新环境治理模式，引入社会资本，按照“宜耕则耕、宜牧则牧、宜林则林”原则进行恢复治理。发展循环经济，提高资源能源利用率，减少废弃物排放。打造循环经济园区。适时推行阶梯水价。加强重点减排项目督查，加快推进污水处理厂、供热企业脱硫工程建设，力争尽快形成减排能力。严格执行排污费征收新标准，实现排污收费全程信息化管理。

(九)实施生活品质提升工程。着力保障最基本民生，扩大普惠性民生，发展高水平民生，满足群众日益增长的多层次、多样化需求。促进更高质量就业。深入实施“促进30周岁以下高校毕业生就业工程”，城镇新增就业1.4万人，城镇登记失业率控制在4.5%以内。组织开展订单式和储备式培训，就业技能培训1.2万人，创业培训2500人。提升公共服务水平。统筹各类教育均衡发展，推进面向全体学生的素质教育。加快教育人才培养，实施校长教师素质提升计划。推进校园“三化”，全市城内学校和乡镇学校全部实现合理硬化。开展防火、防震和交通安全知识进课堂，提高青少年应急避险能力。大力发展职业教育，培养经济社会发展所需人才。深化医药卫生体制改革，推进县级公立医院综合改革试点，引进社会资本参与公立医院改制和经营，提高基层中医药服务能力。落实人口计生奖励政策。加大文化惠民力度，提升文化广场活动档次，引导文化大院规范发展。广泛开展全民健身活动，推进市全民健身中心工程建设，体育馆开工，体育场投入使用。实施社区建设“双百”示范工程，全市城市社区达标率和农村社区建设启动率分别达到88%、85%以上。加大社会保障力度。实施机关事业单位养老保险制度改革。推进医疗保险市级统筹，逐步统一全市医保政策。启动全市城镇居民大病医疗保险工作。适时将机关和参公管理事业单位纳入工伤保险范围。完善保障制度，做好城乡低保提标工作。积极申报省级残疾人就业示范基地项目，全力做好0-6岁残疾儿童免费康复。维护社会安全稳定。完善安全生产责任体系，强化监督管理，严格责任追究。加强市场监管，开展“市场环境优化年”和“质量黑河年”活动。推进食品药品监管体制改革，完善食品药品技术支撑体系。落实“谁执法、谁普法”责任制，深入推进普法工作。健全信访工作机制，建立涉法涉诉信访依法终结制度。做好社区矫正工作。加强国防后备力量建设，加大军地深度融合力度，维护边境秩序稳定。争创全国双拥模范城“三连冠”。

各位代表，为政之要，善谋长远之策，多行利民之举。今年，我们要在全面推进经济社会发展的同时，全力以赴抓好牵动发展大局的“十件大事”，统筹财力办好增进民生福祉的“二十件实事”。

——十件大事。(1)全力争取黑龙江大桥、跨江空中索道项目开工。(2)五大连池民用机场及附属工程上半年开工。(3)加强黑河四岛保护开发，推进黑河水利防护工程。(4)实施好三江治理工程，提高黑河城区段堤防建设标准。(5)抓好黑河城区沿江公园改造。(6)办好第六届中俄文化大集和中俄双子城展览会。(7)力争天然林保护纳入国家政策支持。(8)做好国家级跨境经济合作区谋划申报工作。(9)推进北五铁路建设和北黑铁路改造升级。(10)启动黑河口岸货运码头搬迁工作。

——二十件实事。(1)改善居住条件，市区维修改造90栋老旧住宅楼房，提升改造30个旧住宅小区。两年内基本完成烧火楼、城中村改造任务。(2)改善生活环境，市区撤并、改造锅炉房5个，完成全部拆除改造任务。改造老旧供热管网16.17公里。对老旧铺装人行步道及70处无单位管理的检查井进行维修改造。(3)在黑河城区和具备条件的县(市)启动燃气入户工程，为居民提供更经济、环保、方便的能源服务。(4)倡导“绿色出行”，市区公共自行车站点增至90处，自行车增至2530辆，提高便民服务水平。(5)打造“亲水平台”，结合黑龙江江堤改造工程，改造提升市区西兴路广场、迎恩路广场、邮政路广场、公园路广场、世纪广场，增设下江通道，新建跨江索桥，营造观赏空间，满足市民亲水休闲需求。(6)提高医疗服务水平，开工建设2.3万平方米的市第一人民医院改扩建工程和儿科中心项目。(7)释放政策红利，边民互市贸易交易点投入使用，加大宣传力度，增发边民证5000本。(8)继续推行“阳光早餐”，提高市区一线街路环卫保洁人员工资标准。(9)建设城市“天眼”工程，市区新建高清监控点2198个、无线监控点500个，重点要害区域、场所、治安卡口全覆盖。(10)实施“菜篮子工程”，支持爱辉区1500万元，新建改造标准化大棚450栋、日光节能温室50栋，新增蔬菜裸地种植面积1700亩，地产蔬菜自给率由30%提高到60%以上，逐步提高本地蔬菜供应比例，让市民吃上放心菜。(11)加大保障性安居工程建设力度，全市改造棚户区3146户，农村泥草(危)房3700户。(12)提升教育教学标准，全市改扩建公办幼儿园9所，创建市级一类幼儿园6所，新创建省级义务教育标准化学校8所、先进校3所。(13)加快教育信息化建设，农村和城镇学校接入宽带比例分别达到80%和100%，普通教室多媒体教学设备普及率分别达到50%和80%，30%以上农村学生、50%以上城镇学生和50%以上农村教师、70%以上城镇教师建立个人网络学习空间。(14)建设农村饮水安全工程，全市新建90处，解决5万人饮水不安全问题。(15)建设交通信息服务网络，依托全国“12328”交通运输服务监督电话建设工程，建设覆盖全市范围的交通信息服务中心并投入使用，24小时受理求助、咨询、投诉工作，解决叫车难、出行难问题。(16)关注弱势群体，全市困难群众救助对象住院自付费用救助比例由65%提高到70%，封顶线提高到3万元。殡葬惠民补贴由低保人群扩大到城乡低收入困难居民。(17)提高保障水平，新型农村合作医疗人均筹资标准由390元提高到470元，人均基本公共卫生服务经费由35元提高到40元。(18)守护“舌尖上的安全”，开展全市早市及校园周边食品安全治理专项行动，实施证卡管理，规范经营行为。(19)加快养老机构建设，全市每千名老人拥有养老床位达到30张。(20)实施开发式扶贫，全市完成14个贫困村整村推进项目建设，减少贫困人口5230人。

三、加快建设法治政府

贯彻依法治国方略，加快建设职能科学、权责法定、执法严明、公开公正、廉洁高效、守法诚信的法治政府。 强化科学决策。主动接受人大法律监督和政协民主监督，认真办理人大建议、政协提案，广泛听取民主党派、工商联、无党派人士和人民团体意见、建议。健全科学民主决策机制，完善公众参与、专家论证、风险评估、合法性审查和集体讨论决定等政府决策程序。建立政府法律顾问制度，防范和化解法律风险。深化政务公开，梳理公布政府部门权力清单，提高政府工作透明度。 转变政府职能。继续推进简政放权、放管结合，进一步取消和调整行政审批事项，深化工商登记制度改革，加快推进非行政许可审批事项清理，确保放到位、管得好。深入推进“两集中、两到位”改革，所有非税收费集中到行政服务中心统一收缴。建设网上政务服务中心，逐步实现所有审批事项网上办理。建立公共资源交易中心，实现统一监管。加快社会组织培育，支持行业协会发展，激发社会组织活力。 坚持勤政为民。巩固党的群众路线教育实践活动成果，强化宗旨意识，增强服务群众的能力和水平，打造服务型政府。加强和改进调查研究，深入基层和群众解决实际问题。完善民生工作联席会议制度，畅通民意表达渠道，健全重大决策听证制度，提高公众对政府工作参与度。坚持依法行政，加强对关系群众切身利益执法行为的监督，严格执行重大执法决定法制审核备案制度，维护群众合法权益。 严格廉洁自律。坚持清廉从政、从严治政，认真履行党风廉政建设“一岗双责”，大力推进惩防体系建设，建立完善长效机制。重点在财政资金分配使用、国有资产监管、政府投资、政府采购、公共资源转让、公共工程建设等方面，强化内部流程控制，加强审计监督，防控廉政风险。厉行节约，反对浪费，严控“三公”经费支出，加强财政支出资金绩效考核。严厉整治不作为、乱作为，加大行政问责力度，营造良好发展环境。

各位代表，新形势催生新动力，新常态呼唤新作为。让我们在省委、省政府和市委坚强领导下，紧紧依靠全市人民，用实干和韧劲顶住下行压力，用智慧和创新破解发展难题，用责任和担当增进民生福祉，圆满完成各项工作任务，为黑河更加美好的明天而努力奋斗!
